# Management of adrenal incidentaloma: the role of adrenalectomy may be underestimated

**DOI:** 10.1186/s12893-016-0154-1

**Published:** 2016-06-08

**Authors:** Yun-lin Ye, Xiao-xu Yuan, Ming-kun Chen, Yu-ping Dai, Zi-ke Qin, Fu-fu Zheng

**Affiliations:** Department of Urology, Sun Yat-sen University Cancer Center, State Key Laboratory of Oncology in South China, Collaborative Innovation Center for Cancer Medicine, Guangzhou, Guangdong 510060 China; Department of Urology, The First Affiliated Hospital of Sun Yat-Sen University, Guangzhou, Guangdong 510080 China; Department of Urology, Jiangmen Central Hospital, Jiangmen, Guangdong 529030 China; Department of Urology, The Third Affiliated Hospital of Southern Medical University, Guangzhou, Guangdong 510630 China

**Keywords:** Adrenal tumor, Incidentaloma, Adenoma, Adrenalectomy

## Abstract

**Background:**

To demonstrate clinical characteristics of adrenal incidentaloma in South China and explore its comprehensive management.

**Methods:**

The clinical data of patients with adrenal neoplasm from Jan 1998 to Dec 2012 were retrospectively analysed. Patients with suspicion of adrenal abnormalities or those in whom adrenal abnormalities were detected in the staging procedures of other cancers were excluded. Most patients with adrenal incidentaloma chose to have adrenalectomy, and some chose surveillance. The relationships between clinical features were analysed with a chi-square test and rank sum test.

**Results:**

In total, 634 patients with adrenal incidentaloma were studied. Their age ranged from 17 to 85 years old with a median age of 50 years. Of 478 cases with pathological results, adenoma was the most common tumour (233/478), with 84 cases of pheochromocytoma and 36 cases of adrenocortical carcinoma were 84 and 36. When the tumour size was ≤4 cm, >95 % were benign; when the tumour size was >6 cm, 33 % were malignant. For patients with a tumour size ≤4 cm, 249/376 cases had an adrenalectomy performed. Due to anxiety over a potential malignant transformation and enlargement, most patients (>80 %) under surveillance preferred to undergo adrenalectomy.

**Conclusions:**

Pheochromocytoma and adrenocortical carcinoma were not rare tumours of adrenal incidentaloma, and 4 cm is a good size cutoff to use in the diagnosis of an adrenal incidentaloma. Other than surveillance, laparoscopic adrenalectomy may become the method of choice for management of small adrenal incidentaloma.

**Electronic supplementary material:**

The online version of this article (doi:10.1186/s12893-016-0154-1) contains supplementary material, which is available to authorized users.

## Background

As imaging techniques evolve, the incidence of adrenal incidentaloma is increasing. The reported incidence of adrenal incidentaloma in an autopsy series is 2.1–8.7 %, which was shown to increase with age [1, 2]. However, the diagnosis of adrenal incidentaloma is not often made during a general health screening. Moreover, given the various types of adrenal neoplasm, there are challenges in the management of adrenal incidentaloma.

As laparoscopic adrenalectomy becomes popular, the rate of surgery is increasing for adrenal incidentalomas [3]. For instance, adrenalectomy is recommended for subclinical Cushing’s syndrome, which is related to long-term metabolic disorders [4]. However, approximately 80 % of adrenal incidentalomas are benign and non-hypersecreting. In most reports, nonfunctioning adenoma is the most common tumour, but adrenocortical carcinoma (ACC) plays an important role in some medical centres [3, 5]. In addition, the evaluation of malignant lesions is uncertain, and patients under surveillance suffer psychological burden. Therefore, surgery or a more conservative approach is controversial for adrenal incidentaloma in clinical practice.

In 2002 and 2009, the NIH and AACE/AAES, respectively, published clinical practice guidelines for incidentaloma, recommending follow-ups for patients with small tumours (<4 cm) without malignant characteristics or hormonal activity [6, 7]. However, the multidisciplinary management of adrenal incidentalomas was not diligently performed as guidelines recommended, and several patients were not evaluated by endocrinologists nor did they have an endocrine examination [8]. This study retrospectively analysed the clinical characteristics and management of patients with adrenal incidentalomas, who were referred to the First Affiliated Hospital of Sun Yat-sen University from 1998 to 2012. In South China, this hospital is the largest medical centre with sophisticated endocrinologists and urologists, thus, this study represents the profile of adrenal incidentaloma in South China.

## Methods

### Study design

The clinical data of patients with adrenal neoplasm from Jan 1998 to Dec 2012 were retrospectively analysed. Patients with suspicions of adrenal abnormalities or those in whom adrenal abnormalities were detected during staging procedures of other cancers were excluded. The pathology results and clinical data, including age, presentation, endocrine examination, images and treatment, were retrieved and recorded by an experienced pathologist.

### Treatment

Biochemical evaluations were performed in most patients with an adrenal mass. The first laparoscopic adrenalectomy for adrenal incidentaloma was performed in August 1998; then, for patients with a tumour ≤4 cm, surveillance or laparoscopic adrenalectomy was suggested. For patients with a suspected malignant tumour (enhancement >10 HU or heterogeneous) or with a tumour size >4 cm, open adrenalectomy was considered. For patients in whom there were differing opinions about appropriate management, treatment approaches were not performed according to the guidelines.

### Statistical analysis

Using SPSS 16.0 Package (SPSS, Inc., Chicago, IL), relationships between clinical features, treatment approach and pathological result (malignant or benign) were analysed with the chi-square test and rank sum test; *P* < 0.05 was considered to be significant.

## Results

### Clinical characteristics

During 1998 and 2012, 877 patients with adrenal neoplasm were treated in the First Affiliated Hospital of Sun Yat-sen University, and 634 of them had adrenal incidentalomas. Their age ranged from 17 to 85 years old with a median age of 50 years. Of these, 293 were male, and 341 were female. Then, 138 were followed by surveillance with a median time of 8 months (3–96 months), while an enlargement of the tumour (>1 cm per year or >4 cm) or hormone transformation was detected in 118 patients who chose adrenalectomy. The other 20 patients still preferred surveillance, though 2 of them had tumours that transformed to Cushing syndrome, 1 transformed to pheochromocytoma, and 2 had tumours >4 cm in size (Additional file 1).

### Endocrinologic and pathologic results

With the combination of endocrinologists and urologists, hormonal examination was regularly administered in 529 patients. Of these patients, 69 cases were diagnosed as having pheochromocytoma, 16 with hypercortisolism, 6 with subclinical Cushing syndrome, 13 with aldosteronism, 1 with adrenocortical hypersecretion, and 424 with having a nonfunctional tumour. Pathologic diagnosis was available in 478 patients, and details are listed in Tables 1 and 2.

**Table 1:** Clinical characters of patients without pathological results

	No.	Gender	Age (year)	Size (cm)	Clinical type
		Male: Female	Median (range)	Median (range)	Benign: Malignant
Hormone active	9	5:4	51 (32–75)	7.8 (1.1–15)	3:6
Non-function	104	54:50	55 (25–85)	2 (1.0–14)	92:12
Undefined	43	24:19	62 (31–80)	2.2 (1.0–14)	39:4
Total	156	83:73	56 (25–85)	2.1 (1.0–15)	134:22

**Table 2:** Clinical characters of patients with pathological results

	No.	Gender	Age (year)	Size (cm)	Operation
		Male: Female	Median (range)	Median (range)	Laparoscopic: Open
Malignant pheochromocytoma	9	5:4	47 (27–72)	6 (4.7–8.5)	2:7
Adrenocortical carcinoma	36	17:19	49 (25–77)	9.5 (3.0–17.5)	6^b^:30
Metastasis	5	4:1	59 (53–62)	7.5 (5.0–8)	1^a^:4
Other malignant	7	2:5	41 (20–77)	2.8 (1.0–18)	147:86
Myelolipoma	24	9:15	54 (22–75)	4.5 (1.5–12)	15^a^:9
Ganglioneuroma	22	15:7	37 (21–63)	5.5 (1.1–12)	8:14
Pheochromocytoma	75	39:36	47 (21–77)	5.5 (1.2–23)	23:52
Cyst	32	11:21	37 (22–64)	6 (2.0–18)	25:7
Nodular hyperplasia	12	4:8	43 (36–65)	2 (1.1–3)	11:1
Other benign	23	8:15	40 (21–68)	5.4 (2.0–13.5)	11:12
Total	478	212:266	48 (17–77)	4 (1.0–23)	250:228

^a^Including 1 patient performed fine-needle biopsy other than dissection

^b^All patients performed fine-needle biopsy other than dissection

Following a preoperative endocrine examination, of 233 patients with cortical adenoma, 15 cases were diagnosed as having pheochromocytoma, 10 had hypercortisolism, and 5 had subclinical Cushing syndrome. Of 84 patients with pheochromocytoma (malignant and benign), 48 cases were diagnosed as pheochromocytoma, 1 with aldosteronism, and the others were nonfunctioning.

### History of tumour

In patients with pathologic results, there were 63 patients with a history of benign tumour and 24 with a history of cancer. In these patients with a history of benign tumour, 15 patients had a history of a thyroid tumour, 15 had a history of a hysteromyoma, and 3 had a history of two benign tumours. Of these 63 patients, 4 had adrenocortical carcinoma, and 5 had pheochromocytoma. In patients with a history of cancer, 17 of them had a benign adenoma, 2 had pheochromocytoma, 1 had adrenocortical carcinoma, and 4 had metastasis, including 2 from the liver, 1 from a kidney and 1 from the colon.

For patients without a history of any tumour, 31 had adrenocortical carcinoma, 9 had malignant pheochromocytoma, 1 had metastasis whose primary lung cancer was detected after adrenalectomy, and 6 others were malignant. Of these benign tumours, 67 were diagnosed as pheochromocytoma.

### Tumour size and type

Tumour size was related to its type. In this study, for a tumour size >4 cm or ≤4 cm, the proportion of having a malignant tumour was 56/229 and 1/249, respectively (*P* < 0.001). For a tumour size >6 cm or ≤6 cm, the proportion of having a malignant tumour was 48/144 and 9/334, respectively (*P* < 0.001).

**Fig. 1:**
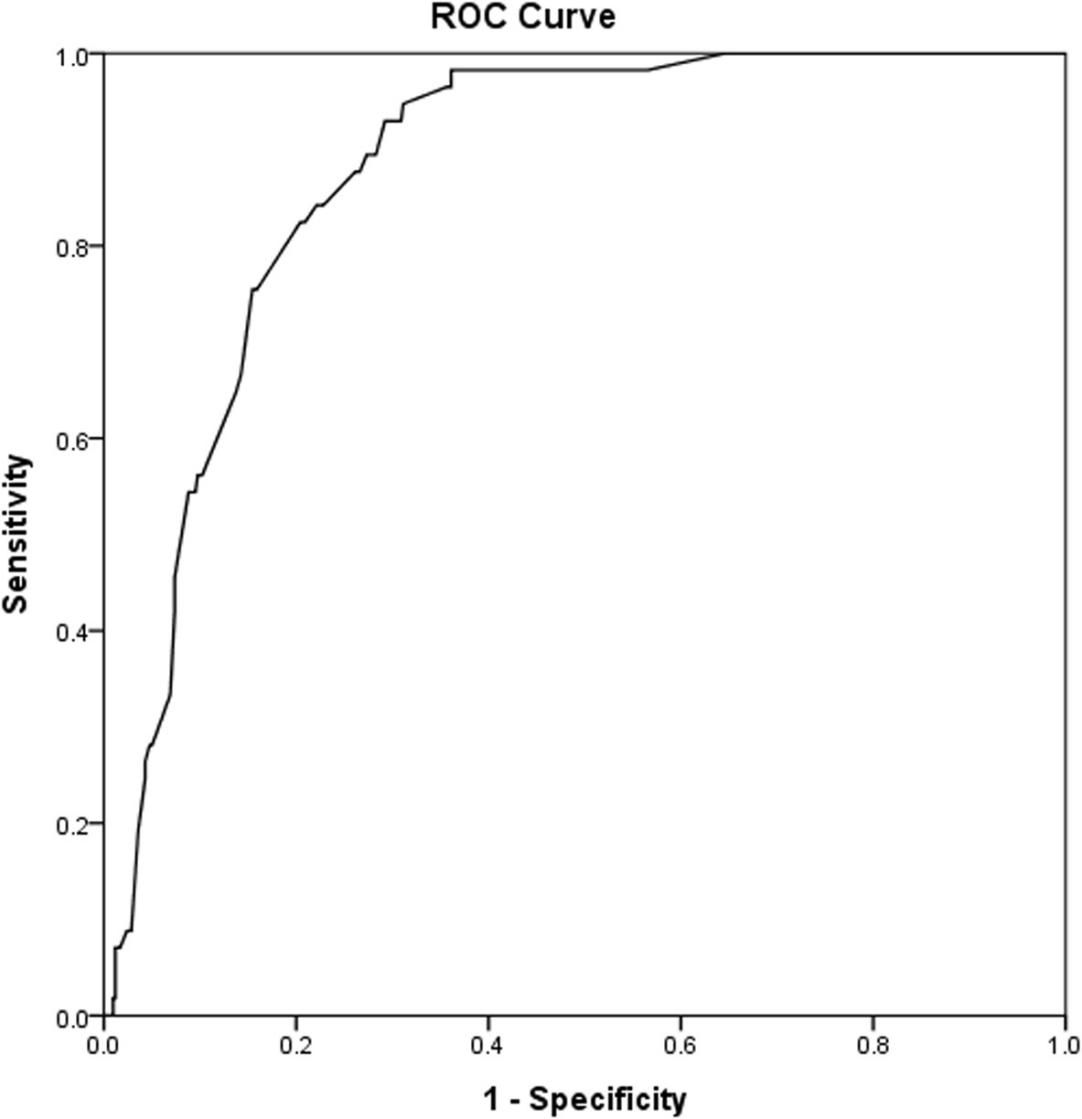
ROC curve of tumour size and tumour type

Using data from patients with pathologic results, a ROC curve was performed, and the cut-off size was 5.4 cm, and the AUC was 0.877 (95 % confidential intervals: 0.84–0.913) with a sensitivity of 93 % and a specificity of 71 % (as shown in Fig. 1). For tumour size >5.4 cm or ≤5.4 cm, the proportion of having a malignant tumour was 53/302 and 4/176, respectively (*P* < 0.001).

### Treatment approaches

Of these 634 patients, 470 had surgery performed, including 228 cases of open surgery and 242 of laparoscopic adrenalectomy, and 8 patients had fine-needle biopsy performed; the other 156 patients chose conservative treatment. The relationships between treatment approach and clinical characteristics are shown in Table 3, and the tumour size of patients who chose laparoscopic adrenalectomy or surveillance was smaller than patients who had open adrenalectomy performed (*P* < 0.001). In all, of 376 patients with a tumour size ≤4 cm, 249 chose adrenalectomy, including 186 cases of nonfunctioning tumours and 23 patients without biochemical evaluation. Of 258 patients with a tumour size >4 cm, 29 refused adrenalectomy, including 8 patients with multi-organ metastasis. For patients who chose conservative treatment, 40/85 patients >55 years were advised to have surveillance for their comorbidities, which included chronic obstructive pulmonary disease, chronic renal failure, severe pneumonia,diabetes with multi-organ involvement,coronary heart disease,hepatocirrhosis with ascitic fluid and severe malnutrition.

**Table 3:** Relationships between treatment approach and clinical characters

	No.	Gender	Age (year)	Size (cm)	Tumor type
		Male: Female	Median (range)	Median (range)	Benign: Malignant
Laparoscopic	242	101:141	52 (20–75)	3 (1.0–18)	239:3
Open	228	102:126	47 (17–77)	6 (1.0–23)	181:47
Surveillance	156	83:73	56 (25–85)	2.1 (1.0–15)	134:22^a^
Biopsy	8	7:1	51 (26–61)	8.5 (7.0–12)	1:7
Total	634	293:341	50 (17–85)	3.2 (1.0–23)	555:79

^a^Clinical tumor type

## Discussions

As popular as abdominal imaging examination has become, adrenal incidentaloma has now become a common finding in clinical practice. Although most cases are benign and nonfunctioning, the presence of an adrenal incidentaloma are still critically concerning for patients and physicians alike because of the risk of transition to malignancy or hormonal hyperfunction. In most reports, malignant and functional lesions represented a small part of adrenal incidentalomas, but some medical centres demonstrated that adrenal adenocarcinoma and pheochromocytoma accounted for 12 % (47/380) and 11 % (42/380), respectively [5]. During a long-term follow-up, the 2-year and 5-year risk of tumour growth and hormonal alteration for adrenal incidentalomas was 29 % and 47 %, respectively [9]. In this study, malignance and pheochromocytoma represented 27.6 % of all patients with pathologic results. Of patients under surveillance, more than 80 % (123/138) had to receive an operation. Meanwhile, several reports recently revealed that for patients with adrenal incidentaloma and subclinical Cushing syndrome, adrenalectomy led to a better outcome than conservative treatment in terms of reducing hypertension and metabolic disorders [10–14]. Therefore, adrenalectomy was recommended to patients with potential of having hormonal alteration or malignant transformation.

As reported, a 4 cm cutoff was associated with a sensitivity of 93 % and a specificity of 24 % in preoperative diagnosis [15]. Additionally, 4.75 cm was reported to be a cutoff with a sensitivity of 90 % and a specificity of 58 % in a minor population [16]. Our cutoff analysis demonstrated that 5.4 cm was a comparable cutoff size with a sensitivity of 93 % and a specificity of 71 %, respectively. Meanwhile, the sensitivity and specificity for cutoffs of 4 cm, were 98 % and 59 %, respectively. In our opinion, for the potential consequences of a misdiagnosis of a malignant tumour, a high sensitivity was preferred. Therefore, we confirmed that 4 cm was a good cutoff, as only 1 tumour size ≤4 cm was malignant in this study. Occasionally, small adrenal cancers were reported, and in this study, the smallest malignant tumour was 3 cm in size.

For patients with pathologic pheochromocytoma, a preoperative evaluation revealed that approximately 43 % patients with pheochromocytoma were not diagnosed before their operations, which was similar to other reports [15, 17]. Therefore, before adrenalectomy for adrenal incidentaloma, anaesthesiology preparation for pheochromocytoma should be performed carefully. Also, 10/19 patients with a tumour ≤4 cm had laparoscopic adrenalectomy performed, and 1 patient was transformed to an open approach due to severe hypertension. As reported, regular preoperative workup could reduce perioperative morbidities [18].

For patients with adrenocortical carcinoma, 7 had metastasis, and 1 had a malignant embolus. The median size was 9.5 cm with range from 3 to 17.5 cm, which was larger than benign tumours (9.5 vs 3.0 cm, *P* < 0.01) and was similar to other reports [19]. Of these patients, 6 patients with metastasis had a biopsy performed, and the others had an open adrenalectomy performed, including 1 patient who chose follow-up for 2 years (with tumour growth from 3 cm to 9 cm); therefore, operative indications were critically important, and a new model for preoperative evaluation might be a supplement to the current guidelines [20].

However, for those patients with benign and nonfunctioning tumours, adrenalectomy may be overutilised, especially in the era of laparoscopic adrenalectomy [3]. Vice versa, surveillance may miss some “silent” tumours with the risk of being hypersecretory or malignant. It was reported that conservative treatment led to a good quality of life, and it was feasible [21]. In our hospital, a conservative approach was presented for patients with nonfunctioning tumours whose diameter was <4 cm and had weak enhancement in imaging examination. However, likely over concern for malignant lesions or hormonal transformation, in actuality, >50 % (470/634) of patients preferred adrenalectomy to remove the tumour and to confirm the pathology results. Of 332 patients with nonfunctioning tumours with a size of ≤4 cm, 209 chose to have adrenalectomy, most of which were performed in recent 5 years. Of 29 patients with a tumour size >4 cm who chose surveillance, only 14 patients refused operation, and the others had an unresectable status. For different psychological and financial reasons, 6 patients with subclinical Cushing’s syndrome and 2 with aldosteronism refused to have adrenalectomy. Psychologically, patients were prone to adrenalectomy.

What’s more, delayed treatment might preclude the opportunity for surgery when needed for ageing patients with comorbidities. In this study, nearly half of patients >55 years had to choose surveillance for poor functional status. As popular as the laparoscopic technique is, this minimally invasive approach has soon replaced traditional adrenalectomy. For diagnostic efficiency of these various adrenal tumours, surgeons tend to want to perform this new convenient operation, remove the tumour, and obtain a pathological result. Moreover, a pathologic result would possibly relieve the psychological and financial burden. Additionally, recurrent evaluation with CT or hormonal examination and close follow-up might not be cost-effective, though no economic analysis was performed. This may play a role in increasing the need for adrenalectomy now, more than in the era of open adrenalectomy. In recent reports, several researchers demonstrated that patients with adrenal incidentaloma were at risk of tumour growth and hormonal transformation and might benefit from adrenalectomy for reduced hypertension, cardiovascular risk and other consequences [11–14, 22, 23]. In a study with a long-term follow-up, cardiovascular events occurred in 22 of 206 patients, and blood pressure control worsened in 34 % patients. Interestingly, a tumour size >2.4 cm was associated with patients developing subclinical hypercortisolism [22]. Therefore, adrenalectomy may be an option for patients with small nonfunctioning adrenal incidentaloma, though it is debatable.

As a well-known medical centre in South China, selective bias was considered as a limitation. Most patients with huge adrenal tumours who transferred from other hospitals had malignancy. Additionally, as a retrospective analysis, patients were enrolled in the urological and endocrine departments without a consistent management protocol, which resulted in different evaluations.

## Conclusions

Nonfunctioning adenoma is the most common tumour of adrenal incidentaloma, and pheochromocytoma and adrenocortical carcinoma were not rare. However, preoperative diagnosis is still undefined, and 4 cm is a good cutoff for diagnosis. Laparoscopic adrenalectomy may become a trend in the operative management of small adrenal incidentaloma.

## Abbreviations

AACE/AAES, American Association of Clinical Endocrinologists and American Association of Endocrine Surgeons; ACC, adrenocortical carcinoma; NIH, National Institutes of Health.

## Additional file

Additional file 1: Table S1. Clinical characters of patients under surveillance who choose surgery. (DOC 37 kb)
