# Supplementary material for: Management of adrenal incidentaloma: the role of adrenalectomy may be underestimated
Source: BMC Surg. 2016 Jun 8;16:41. doi: 10.1186/s12893-016-0154-1 (PMC4898397; doi:10.1186/s12893-016-0154-1)
Supplement: Additional file 1: Table S1. — Clinical characters of patients under surveillance who choose surgery. (DOC 37 kb) [file 12893_2016_154_MOESM1_ESM.doc]

**Table S1 Clinical characters of patients under surveillance who choose surgery**

|  | No. | Gender  Male: female | Age (year)  Median (range) | Operation  Laparoscopic: open | Surveillance time (month) |
| --- | --- | --- | --- | --- | --- |
| Adrenocortical carcinoma | 11 | 4:7 | 50(26-75) | 2^*^:9 | 4 (3-25) |
| Other malignant | 2 | 2:0 | 47-57 | 0:2 | 4 |
| Adenoma | 54 | 26:28 | 48(20-77) | 23:31 | 10 (3-75) |
| Myelolipoma | 9 | 2:7 | 55(38-68) | 7:2 | 3 (3-64) |
| Pheochromocytoma | 13 | 7:6 | 47(21-69) | 2:11 | 12 (3-37) |
| Cyst | 13 | 5:8 | 37(29-63) | 10:3 | 7 (3-24) |
| Other benign | 16 | 7:9 | 41(21-60) | 14:2 | 12 (3-96) |
| Total | 118 | 53:65 | 48(20-77) | 66:52 | 9 (3-96) |

* All patients performed fine-needle biopsy other than dissection.
